# High resolution monitoring of valvular interstitial cell driven pathomechanisms in procalcific environment using label-free impedance spectroscopy

**DOI:** 10.3389/fcvm.2023.1155371

**Published:** 2023-06-20

**Authors:** Julia Böttner, Sarah Werner, Lukas Feistner, Tina Fischer-Schaepmann, Katherina Neussl, Michael A. Borger, Holger Thiele, Petra Büttner, Florian Schlotter

**Affiliations:** ^1^ Department of Cardiology, Heart Center Leipzig at University of Leipzig, Leipzig, Germany; ^²^ Department of Cardiac Surgery, Heart Center Leipzig at University of Leipzig, Leipzig, Germany

**Keywords:** FCAVD, extracellular matrix (ECM), electrochemical impedance spectroscopy (EIS), apoptosis, calcification

## Abstract

**Introduction:**

Fibro-calcific aortic valve disease has high prevalence and is associated with significant mortality. Fibrotic extracellular matrix (ECM) remodeling and calcific mineral deposition change the valvular microarchitecture and deteriorate valvular function. Valvular interstitial cells (VICs) in profibrotic or procalcifying environment are frequently used in vitro models. However, remodeling processes take several days to weeks to develop, even in vitro. Continuous monitoring by real-time impedance spectroscopy (EIS) may reveal new insights into this process.

**Methods:**

VIC-driven ECM remodeling stimulated by procalcifying (PM) or profibrotic medium (FM) was monitored by label-free EIS. Collagen secretion, matrix mineralization, viability, mitochondrial damage, myofibroblastic gene expression and cytoskeletal alterations were analyzed.

**Results and Discussion:**

EIS profiles of VICs in control medium (CM) and FM were comparable. PM reproducibly induced a specific, biphasic EIS profile. Phase 1 showed an initial impedance drop, which moderately correlated with decreasing collagen secretion (*r* = 0.67, *p* = 0.22), accompanied by mitochondrial membrane hyperpolarization and cell death. Phase 2 EIS signal increase was positively correlated with augmented ECM mineralization (*r* = 0.97, *p* = 0.008). VICs in PM decreased myofibroblastic gene expression (*p* < 0.001) and stress fiber assembly compared to CM. EIS revealed sex-specific differences. Male VICs showed higher proliferation and in PM EIS decrease in phase 1 was significantly pronounced compared to female VICs (male minimum: 7.4 ± 4.2%, female minimum: 26.5 ± 4.4%, *p* < 0.01). VICs in PM reproduced disease characteristics in vitro remarkably fast with significant impact of donor sex. PM suppressed myofibroblastogenesis and favored ECM mineralization. In summary, EIS represents an efficient, easy-to-use, high-content screening tool enabling patient-specific, subgroup- and temporal resolution.

## Introduction

Fibro-calcific aortic valve disease (FCAVD) is highly prevalent with a high risk of progression towards heart failure or death, if left untreated (1, 2). Despite increasing insights into FCAVD pathomechanisms, there is no current pharmacological intervention to attenuate or reverse FCAVD, leaving surgical aortic valve (AV) replacement or interventional AV implantation as the only therapeutic options (3). FCAVD is characterized by inflammatory cell infiltration, lipid deposition, fibrotic valve thickening and stiffening, and finally calcific mineralization of the valve leaflets (2, 4–6). These processes stimulate resident valvular interstitial cells (VICs) towards myofibroblastic and osteogenic differentiation. Activated myofibroblastic VICs actively remodel the extracellular matrix (ECM) towards fibrosis, and may evolve towards osteoblastic VIC phenotype that triggers ECM calcification (7). ECM remodeling is characterized by collagen accumulation, proteoglycan degeneration and elastin fiber fragmentation (8–10). These molecular and structural alterations of the AV cause deterioration of valvular function, finally leading to AV stenosis (2). Sex appears to have an impact on fibrosis and calcification, since women tend to have a more fibrotic phenotype while men manifest a more calcific phenotype (11).

*In vitro,* VIC models are characterized by pronounced variability introduced by donor age, sex, comorbidities, degree of stenosis, valvular anatomy (12) and culture conditions (13). Therefore, standardization is required for valid, comparable data acquisition. Three major *in vitro* environments that recapitulate individual aspects of FCAVD have been utilized: transforming growth factor-*β* (TGF-*β*)-dependent profibrotic medium (FM) inducing myofibroblastogenesis (14, 15); osteogenic medium (OM) using glucocorticoid-driven cellular differentiation and expression of alkaline phosphatase to hydrolyze phosphate sources into free phosphate (16–21); and procalcifying medium (PM) (13, 22–24), that mimics a hyperphosphatemic milieu akin to advanced stage chronic kidney disease (25). While OM was shown to have variable calcification success depending on donor and cell culture passages (13, 21), PM generates reproducible calcification (13). Thus, the current study focuses on PM. However, the exact molecular and physiological alterations over time are largely unknown for all three *in vitro* milieus. Analysis of *in vitro* VIC-associated fibrosis and calcification requires a multi-method approach including a variety of analytic tools. Electrochemical impedance spectroscopy (EIS) represents a unique technique, allowing label-free, non-invasive, real-time monitoring of cellular responses to external stimuli (26). Changes in cellular impedance uncover and integrate intra- and extracellular processes, cellular alterations in cell-cell- or cell-matrix-connections (27) and morphological changes during cell death (28), proliferation (29) and differentiation (30). This study characterized VIC-driven ECM remodeling and cellular responses in profibrotic and procalcifying environments using EIS.

## Methods

### Materials

Collagenase (from Clostridium histolyticum), transforming growth factor *β* (TGF-*β*), sodium dihydrogenphosphate (NaH_2_PO_4_), L-ascorbic acid, hexadecylpyridinumchloride, propidiumiodide, FITC-dextran (40 kDa) and JC-10 mitochondrial staining kit from Merck (Darmstadt, Germany), Alizarin Red from Cell Systems (Troisdorf, Germany), OsteoSense 680 EX from Perkin Elmer (Waltham, USA) and Calcein-AM from Thermo Fisher (Waltham, USA) were used for culture media, sample preparation and staining.

### Isolation and culture of human valvular interstitial cells

Human VICs were obtained from 10 patients undergoing surgical AV replacement. The study was approved by the local Ethics Committee (Medical Faculty, University Leipzig, registration number 128/19-ek) and all patients gave written informed consent in accordance with the Declaration of Helsinki. AVs were minced and enzymatically digested in a serum-free collagenase (125 U/ml) solution under steady rotation at 15 rpm at 37°C for a total of four hours. Cells were passed through a 40 µm cell strainer and pelleted by centrifugation. VICs were cultured in high glucose DMEM (Thermo Fisher, #61965059) supplemented with 10% FBS (PAN Biotech, Aidenbach, Germany) and 1% penicillin/streptomycin (Thermo Fisher, Waltham, USA), further termed as growth medium (GM). Cells were cultured in a humidified atmosphere at 37°C and 5% CO_2_ and passaged at 90% confluency. Experiments were performed in passages 3 to 4 at 100% confluence in GM. Control cultures were grown in high glucose DMEM (+5% FBS + 1% penicillin/streptomycin) referred to as control medium (CM). Calcification was induced using PM consisting of high glucose DMEM (+5% FBS +1% penicillin/streptomycin) supplemented with 2 mM NaH_2_PO_4_ (pH 7.4) and 50 µg/ml L-ascorbic acid. Fibrosis was induced using FM containing high glucose DMEM (+5% FBS + 1% penicillin/streptomycin) supplemented with 0.5 ng/ml TGF-*β*.

### Real-time impedance spectroscopic monitoring

Impedance spectroscopy measurements were performed using an ACEA xCELLigence® Real-time Cell Analyzer (RTCA) DP instrument (ACEA Bioscience, San Diego, USA). The RTCA system holds 3 “E-Plate 16” electrode arrays and a computer-based control unit. The E-Plates exhibit gold interdigital electrodes covering 80% surface of each well. 7000 cells were seeded per well and immediately continuously recorded for impedance changes. Values of the cell-free electrodes were set as blank. Rising impedance values indicated increasing electrode coverage, first via cellular adhesion and secondly due to cell proliferation. Constant impedance values represented confluent cellular monolayers. At this stage, CM, FM or PM treatment for a total of 20 days was started. Experimental cellular impedance (CI) values were recorded every 60 min for 20 days. Data were normalized to the last value that was measured before the experiment (*t* = 0). Growth and experimental media were replaced every 2 to 3 days.

### Alizarin Red and near-infrared molecular staining and quantification of calcium-containing mineral deposition

Calcium-containing cellular mineral depositions were quantified using Alizarin Red staining at day 0 and then every other day until day 20 and finally on day 21 in CM, FM or PM. VICs were washed with PBS, fixed with 10% formaldehyde and stained with 2% Alizarin Red for 15 min. The cells were then washed with deionized water and air-dried. Afterwards, hexadecylpyridinumchloride (100 mM) was added and incubated in the dark at room temperature for 3 h. The resulting solution was diluted 10-fold and optical density was measured at 540 nm using a microplate reader (Tecan, Männedorf, Switzerland). Data was normalized to *t* = 0.

For visualization of VIC-driven hydroxyapatite (HA) deposition, a near-infrared, bisphosphonate-based molecular probe (OsteoSense 680 EX) was used. Therefore, 2 nmol OsteoSense 680 EX solution was added to the cell cultures for 24 h and subsequently visualized at 680 nm using a fluorescence microscope (Keyence, BZ-X800). The OsteoSense-stained area was quantified using ImageJ (31).

### Pro-collagen type 1 secretion

Pro-collagen type 1 alpha 1 (pro-COL1A1) secretion was measured in cell culture supernatants using ELISA according to the manufacturer's recommendations (R&D Systems, Minneapolis, USA, #DY6220-05).

### Staining of viable and dead cells

Calcein-AM (2 µM) and propidiumiodide (PI) (3 µM) were added to the VIC cultures at the indicated time points to quantify viable and dead cells. Cells were incubated at 37°C and 5% CO_2_ for 15 min. Viable and dead cells in CM or PM were visualized at 525 nm and 620 nm, respectively, using a microplate reader (Tecan, Männedorf, Switzerland).

### Changes in mitochondrial transmembrane potential

As a hallmark of cell death, mitochondrial transmembrane potential *Δψ*m was determined in PM or CM treated VICs at 0, 3, 7 and 12 days using the “Mitochondrial Membrane Potential Kit” (Merck, Darmstadt, Germany) following the manufactureŕs instructions. A ratio of red-to-green fluorescence was determined expressing the amount of dead cells in relation to viable cells.

### Macromolecular permeability assay

Permeability of the VIC monolayer due to CM or PM treatment was determined according to Bischoff et al. (32) at indicated time points (*t* = 0, 3, 7, 12, 15, 18, 21 days). In brief, VICs were grown on the upper compartment of Transwell inserts (12 mm diameter, 0.4 µm pore size; Greiner BioOne, Kremsmünster, Austria) to confluency. To measure macromolecular transmission through the cell monolayer, 1 mg/ml FITC-Dextran in CM was added to the upper compartment and incubated for 30 min. Then, 50 µl samples from the lower compartment were subjected to fluorescence measurement at 525 nm. FITC-Dextran standard curve ranging from 1000 µg/ml to 0.001 µg/ml in CM was used for quantification.

### Quantitative real time-polymerase chain reaction (qRT-PCR)

Gene expression of ACTA2 in CM- or PM-treated VIC cultures at day 0, 7 and 21 was analyzed using qRT-PCR. Takyon NoRox Sybr Mastermix Blue (Eurogentec, Lüttich, Belgium) on a BioRad CFX system (BioRad, Hercules, USA) was used. Exon-spanning primers were designed with an annealing temperature of 60°C (5´-3´sequences for forward: CGTTACTACTGCTGAGCGTG and reverse: CGATGAAGGATGGCTGGAAC). Standard curves were used to calculate copy numbers and reaction efficiency. Measurements were done in triplicates and specimen with standard deviation > 0.3 were excluded. Hypoxanthine phosphoribosyltransferase 1 (HPRT1, (5´-3´sequences for forward: CTCATGGACTGATTATGGACAGGAC and reverse: GCAGGTCAGCAAAGAACTTATAGCC) as a stable housekeeping gene was used for expression normalization of the target gene.

### Phalloidin staining

Actin cytoskeleton alterations were analyzed using Phalloidin-iFluor 594 reagent (Abcam, Cambridge, UK) at day 0, 7, 14 and 21. Cells were fixed and F-actin fibers were stained with Phalloidin for 60 min at room temperature. Nuclei were stained with 10 µg/ml Hoechst 33342 (Invitrogen, Waltham, USA) for 10 min at room temperature. Visualization was done using a Keyence BZ-X800 microscope.

### Statistical analysis

Statistical analyses were performed using GraphPadPrism 6 (San Diego, USA). Data were checked for normal distribution using Shapiro-Wilks test and are presented as mean ± standard error of the mean (SEM). “n” accounts for the number of biological replicates (derived from different donor valves) in the experiments and is declared for each dataset. ELISA and qRT-PCR raw data were median-normalized, when significant plate-to-plate median differences indicated batch effects.

Group differences were analyzed using one-way analysis of variance (ANOVA) or Student´s t-test. Time traces were assessed by two-way repeated measures ANOVA. Bonferroni correction was used as post-hoc test. All *p*-values <0.05 were considered statistically significant. Pearson's correlation coefficient (r) was calculated to test for associations between data of 2 different techniques.

## Results

### Label-free EIS based real-time monitoring of PM-induced alterations in VICs

**Figure 1:**
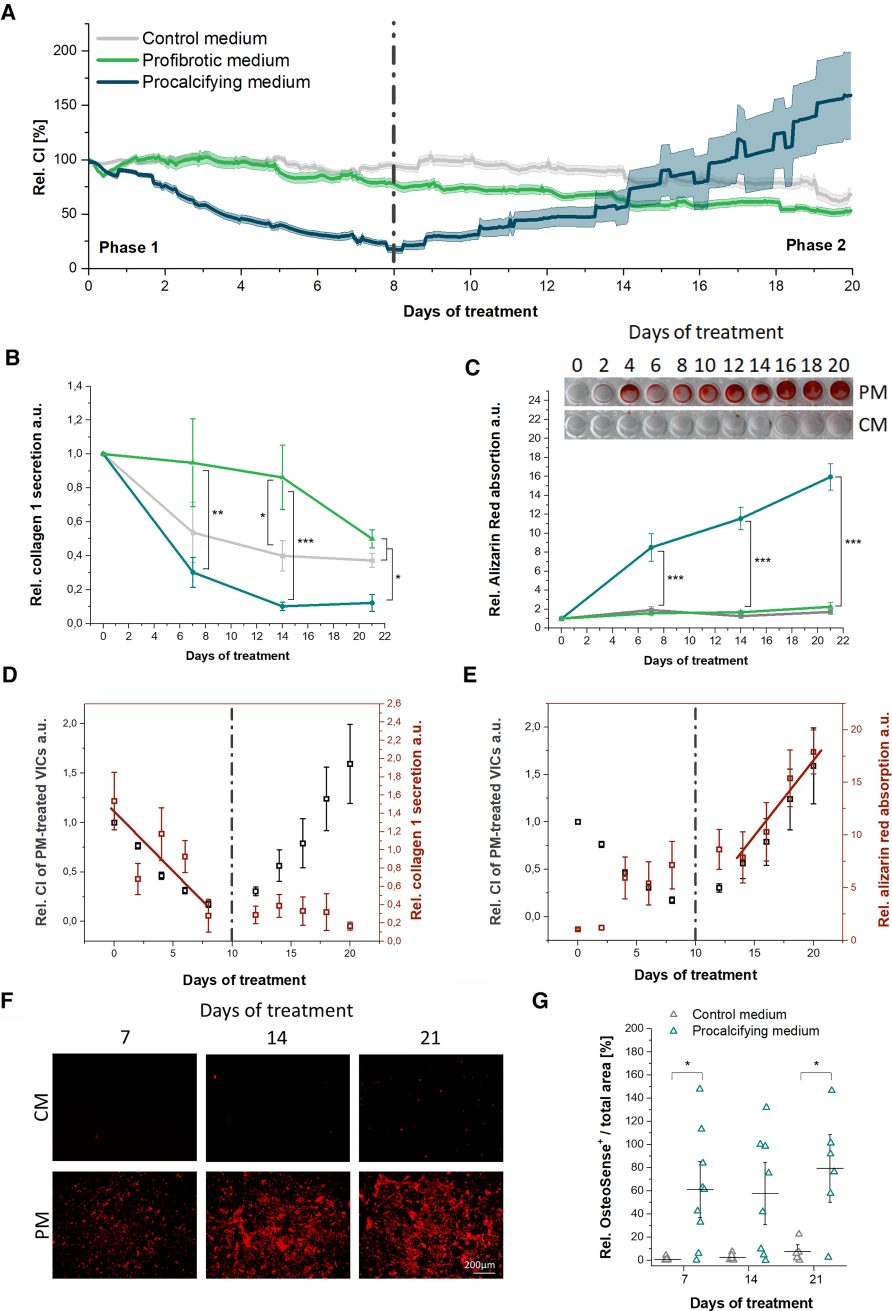
Electrochemical impedance spectroscopy (EIS) real-time monitoring of human valvular interstitial cells (VICs) *in vitro* in profibrotic or procalcifying environment. (**A**) Averaged time traces of VICs cultivated in control medium (CM), profibrotic medium (FM) or procalcifying medium (PM) for 20 days. *n* = 10. Mean ± SEM (SEM shown as continuous lines). (**B**) The relative collagen type 1 secretion and (**C**) Alizarin Red absorption of VICs in CM, PM or FM. *n* = 10. Association plots and Pearson correlation coefficients (r) for EIS and (**D**) collagen secretion or (**E**) Alizarin red absorption for the exact time traces of PM-treated VICs. Pearson correlation coefficient r was calculated for phase 1 (day 0 to 8) and phase 2 (day12 to 21) separately. Visualization of (**F**) near infrared molecular imaging for hydroxyapatite (HA) and (**G**) quantification of the HA-positive area during the 21 day culture period. The dashed lines indicate the inflection point between phase 1 and phase 2. CI, Cellular impedance.

Non-invasive EIS was used to screen PM- and FM-induced cellular responses in VICs (Figure 1A). The EIS signal of VICs in CM was stable for 14 days and then declined to 67.9 ± 5.8% at day 20. In VICs treated with FM, the EIS signal declined slowly from day 5 down to 53.0 ± 3.6% at day 20. Interestingly, in PM, VICs showed a biphasic EIS time profile, which differed significantly from EIS time traces of CM- or FM-treated VICs. PM instantly reduced the cellular impedance signal to a minimum at 17.1 ± 3.3% at day 8 (*p* = 0.039, “phase 1”). During the following 12 days, termed “phase 2”, the signal increased significantly up to a maximum of 159.3 ± 40.1% on day 20 (*p* < 0.001 vs. minimal EIS signal, *p* = 0.14 vs. day 0).

PM in a cell-free environment also had an impact on EIS signals showing gradually increasing signals (see Supplementary Figure S1).

### Impact of collagen secretion and calcification in PM on the biphasic EIS time profile

CM- and FM-treated VIC cultures showed comparable collagen type 1 secretion and comparable, negligible Alizarin Red absorption (Figures 1B,C). PM treatment significantly reduced the collagen type 1 secretion of VICs 3.3-fold after 14 days (*p* = 0.035) compared to CM treatment. PM VICs had significantly reduced collagen type 1 secretion of 3.2- to 8.6-fold compared to FM treated VICs (*p* < 0.05) during the complete observation period and a 3- to 4-fold reduction compared to CM treated VICs (*p* < 0.05) at day 14 and 21 (Figure 1B). Compared to FM- and CM-treated VICs, PM stimulated massive extracellular deposition of calcium-containing mineral material, expressed by 4.5- to 8-fold increased Alizarin Red absorption during the complete observation period (*p* < 0.001, Figure 1C). Pearson correlation coefficients (*r*) were calculated to detect potential associations between the PM-specific EIS signals and the VIC-driven collagen secretion and mineral deposition (Figures 1D,E). As EIS signal was biphasic, *r* was calculated for phase 1 (day 0–8) and phase 2 (day 12–21) separately. A very high negative association was found for the increasing Alizarin Red absorption and the decreasing EIS signal with *r* = −0.95 (*p* = 0.015) in phase 1. Additionally, the EIS signal in phase 1 was moderately correlated to the decreasing collagen type 1 secretion with *r* = −0.67 (*p* = 0.22). A very high, positive correlation of the EIS signal in phase 2 with the increasing Alizarin Red absorption with *r* = 0.97 (*p* = 0.008) was found.

Additional staining of PM-stimulated hydroxyapatite (HA) deposition by VICs using a near-infrared molecular probe confirmed the Alizarin Red results. However a less intense staining was evident due to specificity to HA (Figures 1F,G). Quantification of the OsteoSense-positive area revealed higher HA-deposition of VICs in PM compared to CM.

### Viability and myofibroblastogenesis of PM treated VICs

**Figure 2:**
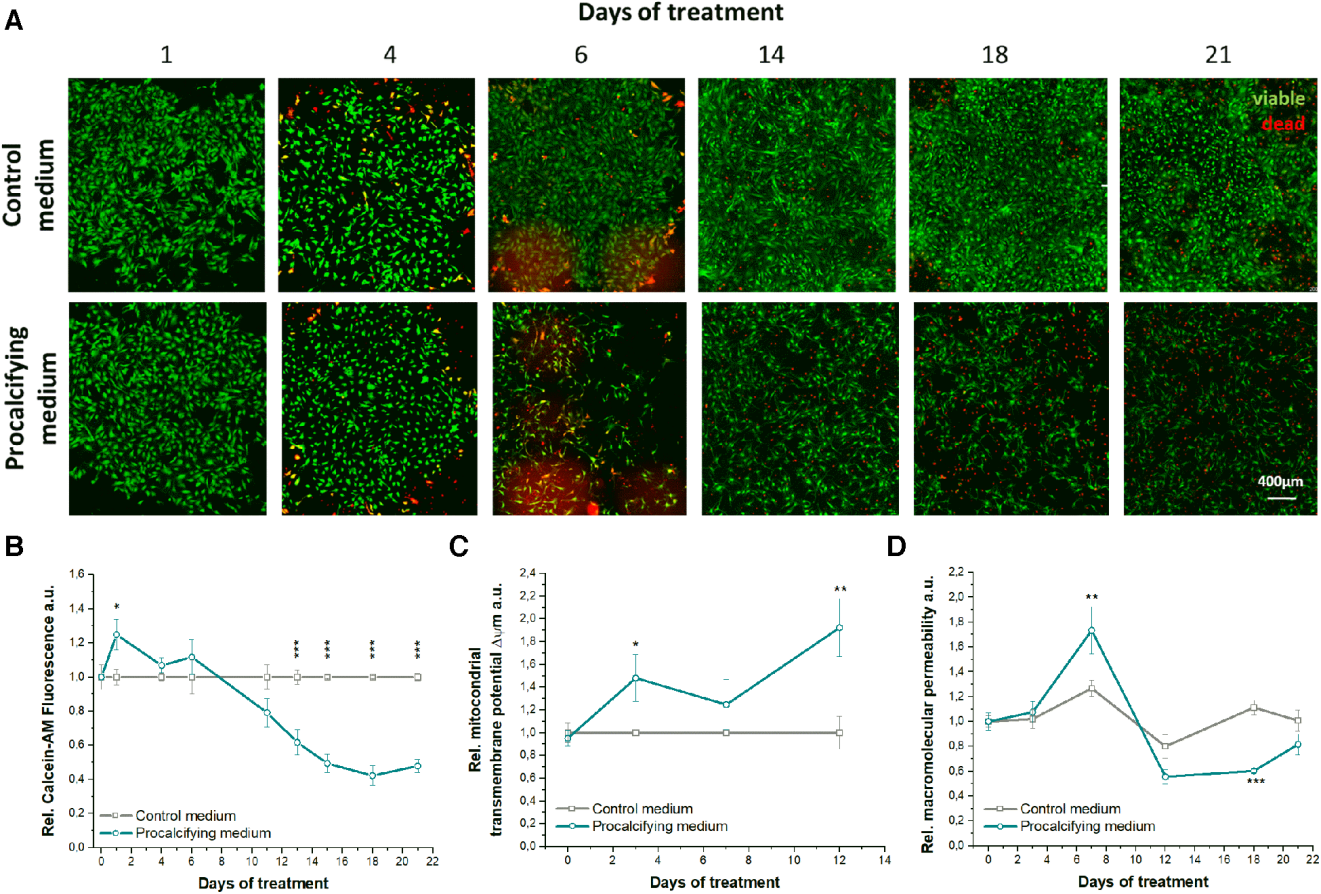
Viability and cell death of VICs in PM. (**A**) Visualization of viable, Calcein-AM + and dead, propidiumiodide + VICs cultured in control or procalcifying medium over 21 days. (**B**) Calcein-AM quantification, (**C**) mitochondrial transmembrane potential *Δψ*m and (**F**) relative macromolecular permeability of VICs in control or procalcifying medium normalized to *t* = 0. Mean ± SEM. *n* ≥ 4.

**Figure 3:**
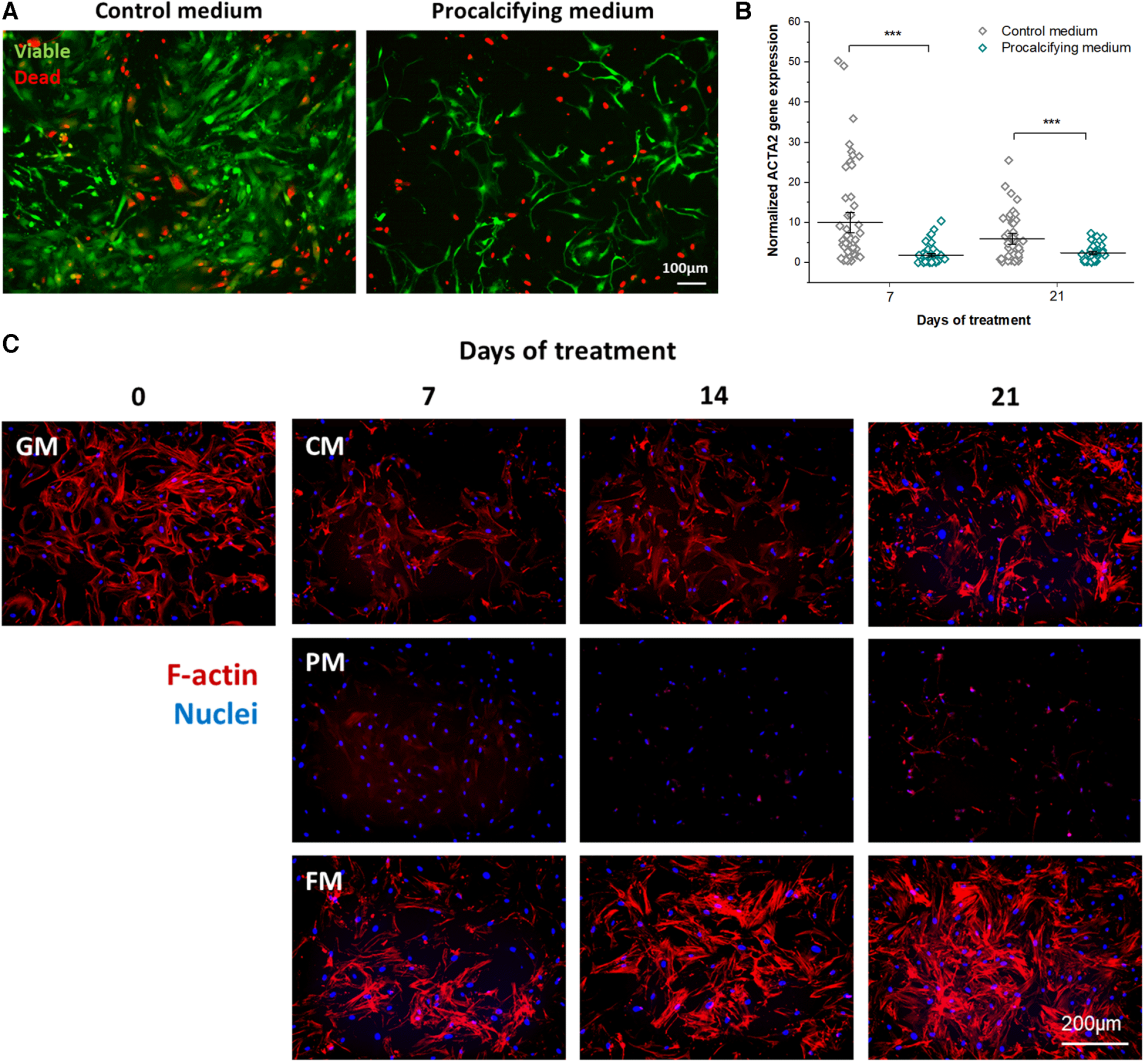
Myofibroblastogenesis of VICs in PM. (**A**) Visualization of viable, Calcein-AM^+^ and dead, propidiumiodide^+^ VICs cultured in control medium (CM) or procalcifying medium (PM) over 21 days. *n* = 5. (**B**) Gene expression of alpha smooth muscle actin (ACTA2) of VICs in CM and PM at day 7 and 21. ACTA2 gene expression was normalized to expression of hypoxanthine phosphoribosyltransferase 1 (HPRT1), *n* = 10. (**C**) Phalloidin staining of F-Actin cytoskeleton modulation of VICs in CM, PM, growth medium (GM) and profibrotic medium (FM). *n* = 3.

The impact of experimental media on cellular alterations, viability and cytotoxicity, and therewith also on the EIS signal, was investigated. Visualization of viable (Calcein-AM^+^, green) and dead VICs (PI^+^, red), respectively, illustrated specific reactions to CM or PM (Figure 2A). VICs in CM maintained round, mostly Calcein-AM^+^ cell clusters during the observation period. Starting from day 4, dead VICs next to and inside the cell clusters with increasing frequency were seen up to 21 days. In contrast, PM-treated VICs formed viable, Calcein-AM^+^ cell clusters only until day 4, when the amount of dead cells (PI^+^) started to increase. From day 6 on, cluster formation was disrupted resulting in a homogeneous distribution of viable and dead VICs. Importantly, the PM Calcein-AM fluorescence signal, indicative for viability, was significantly reduced by 38.3 ± 7.3% from day 13 on up to 52.1 ± 3.9% (*p* < 0.001 vs. CM) at day 21 (Figure 2B). Additionally, the mitochondrial transmembrane potential *Δψ*m of PM-treated VICs revealed significant mitochondrial hyperpolarization up to 48.1 ± 20.8% (*p* = 0.038) at day 3 and 92.3 ± 25.4% (*p* = 0.001) at day 12 compared to CM-treated VICs (Figure 2C). Macromolecular permeability, as a measure of monolayer disruption, of PM-treated VIC monolayers showed a biphasic time trace (Figure 2D). In PM-treated VIC monolayers macromolecular permeability was significantly enhanced up to 1.4-fold at day 7 (*p* = 0.001 vs. CM) and decreased constantly reaching its minimum of a 1.9-fold reduction at day 18 (*p* < 0.001 vs. CM). From day 6 on, VICs in PM had a spindle-like morphology with long and thin cell bodies, whereas VICs in CM exhibited a flattened, rounded morphology (Figure 3A). Based on this observation of morphological alterations, the relative amount of activated VICs in CM and PM was evaluated by analyzing ACTA2 (alpha smooth muscle actin) gene expression as a marker of myofibroblastic activation (Figure 3B). ACTA2 expression in CM was 6-fold higher at day 7 and 2-fold higher at day 21 compared to PM (both *p* < 0.001). Phalloidin staining of F-actin fibers confirmed an abundance of stress fibers of VICs in FM and a lack of stress fiber assembly in PM, which is in line with ACTA2 gene expression (Figure 3C).

### Donor- and sex-specific ECM remodeling in PM

**Figure 4:**
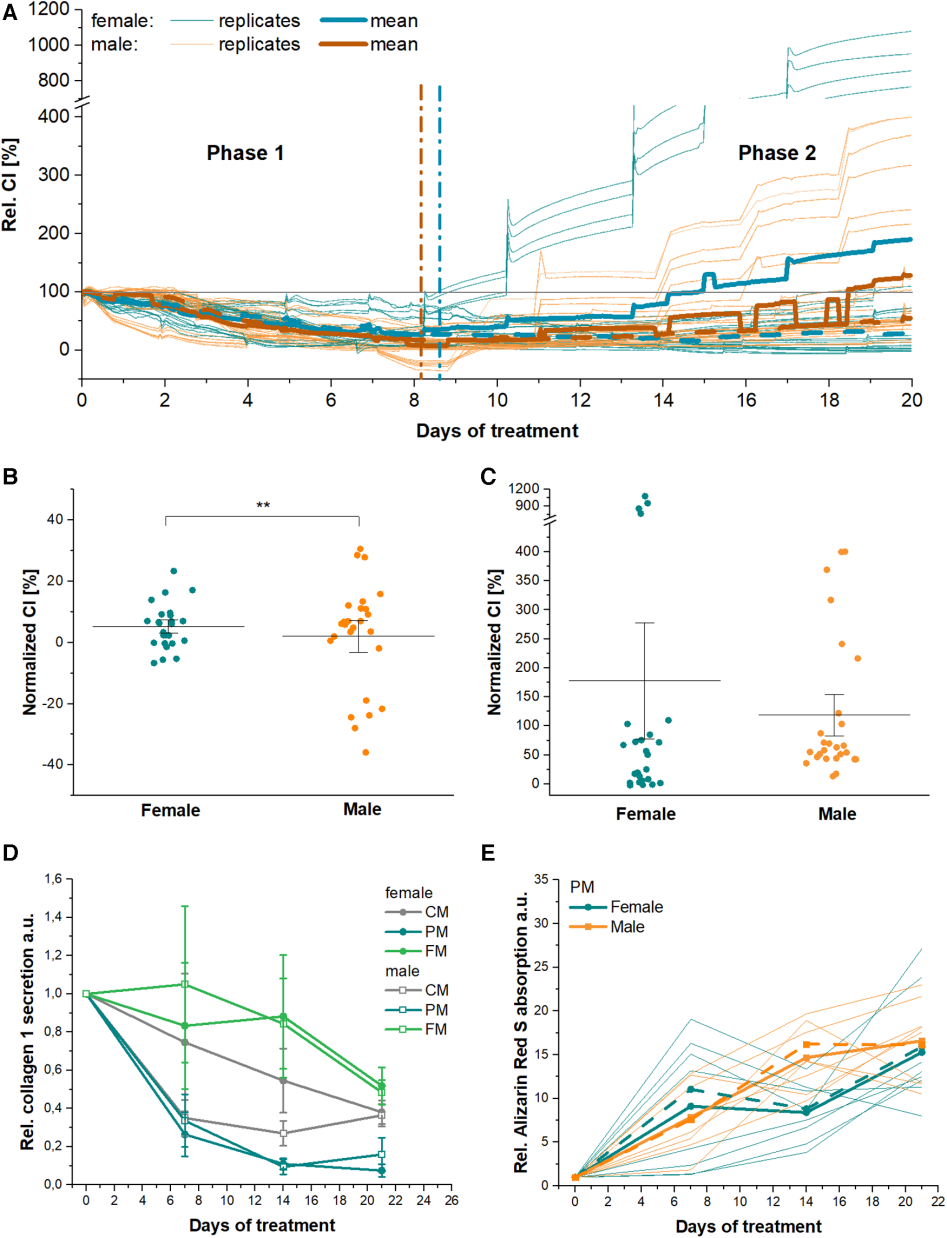
Electrochemical impedance spectroscopy (EIS) analysis of PM-induced sex- and donor-dependent effects in valvular interstitial cells (VICs). (**A**) Single and averaged time traces (bold lines) of either male (orange) or female (petrol) VICs in procalcifying medium PM for 20 days. (**B**) Minimal and (**C**) maximal EIS signals of male and female VICs in PM. Relative (**D**) collagen 1 secretion and (**E**) Alizarin Red absorption data from Figures 1B,C stratified by donor sex. Dashed lines indicate means without the impedance based extreme values (values >90 percentile were excluded). *n* = 5 vs. 5. CM, Control medium; FM, Profibrotic medium; CI, Cellular impedance.

EIS was used to analyze donor- and sex-specific impedance patterns (Figure 4 and Supplementary Figure S2). First, EIS was used to determine the doubling time as a measure of proliferation potential of VICs in GM. On average, female VICs were found to exhibit a 13 ± 4 h longer doubling time than male VICs (*p* = 0.017). In general, the PM-specific, biphasic EIS time profile was observed for all patientś VICs (Figure 4A and Supplementary Figure S3). However, male VICs entered phase 2 significantly earlier at day 8.7 ± 0.4, while female VICs ended phase 1 not until day 11.2 ± 1.0 (*p* = 0.03). In addition, in male VICs the decrease of cellular impedance in phase 1 was significantly more pronounced (minimum: 7.4 ± 4.2%) than in female VICs (minimum: 26.5 ± 4.4%, *p* = 0.003) (Figure 4B). A high donor-specific heterogeneity of EIS phase 1 and 2 characteristics was observed. Seven out of 10 donors showed moderate phase 2 enhancement (+74% to +107% total growth) (Figures 4B,C). One female donor showed lowest phase 2 enhancement (+2.1 ± 0.9%), another female donor showed strongest phase 2 enhancement with a total increase of +914.4 ± 66.7%. In contrast, the strongest male phase 2 enhancement was +349.4 ± 32.7%. Averaged phase 2 EIS time traces of male and female VICs PM were comparable with an increase of +156.1 ± 20.0% and +226.2 ± 61.2% (*p* = 0.80), respectively.

Collagen type 1 secretion (Figure 4D) was comparable from male and female VICs in CM, FM and PM (*p* > 0.05). Alizarin Red staining showed comparable calcium-containing mineral deposition of male and female VICs (*p* > 0.05).

## Discussion

This study investigated pathomechanisms observed in VICs in profibrotic (FM) and procalcific (PM) environments, frequently used FCAVD *in vitro* models, using non-invasive, label-free EIS monitoring. The key findings can be summarized as follows:

PM treatment reproducibly:

- forced a biphasic, specific EIS profile in VICs
- strongly decreased collagen 1 secretion in favor of ECM mineralization
- induced mitochondrial hyperpolarization and reduced VIC viability
- suppressed ACTA2 expression and stress fiber assembly
- revealed donor- and sex-specific inter-individual heterogeneity *in vitro*.

FM treatment:

- maintained collagen type 1 secretion with constant EIS signals
- stimulated stress fiber assembly in VICs.

### PM-specific EIS profile is associated with modulation of ECM components

The observed PM-specific biphasic EIS signal with an initial drop followed by a secondary increase indicates differential electrode coverage during the cultivation period (32). A moderate positive Pearson correlation coefficient between the phase 1 EIS decrease and the declining collagen 1 secretion suggests that suppression of collagen production contributed to the initial EIS decrease. Whether the decline in collagen secretion is due to a direct effect of PM on collagen secretion or secondary to a loss of collagen-producing cells remains to be elucidated. In contrast, in FM EIS as well as collagen secretion were constant over time. Thus, EIS is of limited utility to monitor profibrotic ECM modulation *in vitro*. This is supported by a study of Fuentes-Velez et al. who demonstrated that fibrotic remodeling in hepatic tissue cultures is not associated with EIS alterations (33). But, synergistic effects including the modulation of collagen secretion and beginning ECM mineralization accompanied by major cell loss may contribute to the Phase 1 EIS signal.

Phase 2 EIS increase in PM was correlated with VIC-driven ECM mineralization as confirmed by HA-specific near-infrared molecular probe staining and Alizarin Red staining of calcium-containing mineral deposits (34). It was previously demonstrated that EIS signals increase when HA is formed and decrease when HA degrades (35). HA, carbonated HA and other calcium-, phosphate- and carbonate- containing HA are common organic compounds in AVs from patients with severe AS (36).

In summary, we conclude that the PM-specific EIS phase 2 can be assigned to ECM mineralization. In addition, PM alone showed a slow gradually increasing EIS time trace due to low, passive calcific matter formation on the electrode surface. This is a first hint towards an active acceleration of a passive calcification process by VICs. Since PM in a cell-free environment showed slight passive calcific precipitation, the biphasic EIS time trace only occurred in the presence of VICs and induced ECM calcification in a significantly more pronounced fashion.

Collagen deposition *in vivo* is well-documented to prepare the ground for ECM mineralization of the AV by providing a supportive structure for mineral material aggregation (37, 38). Importantly, PM simultaneously decreased the collagen secretion in favor of ECM mineralization comprehending FCAVD pathobiology *in vivo* (5, 39).

### PM-induced VIC-driven ECM mineralization associates with phosphate-driven apoptosis

As mentioned above, the moderate correlation between EIS and suppression of collagen secretion might not solely explain the initial phase 1 EIS drop. Hence, intracellular responses of VICs to PM treatment were analyzed as well. Phase 1 EIS was characterized by a decrease of viable VICs due to mitochondrial hyperpolarization and apoptosis-mediated cell loss, which was accompanied by a high macromolecular permeability illustrating the disruption of the VIC monolayer integrity. Hyperpolarization of the mitochondrial membrane potential is a hallmark of apoptotic cell death and can be induced by high extracellular inorganic phosphate (40) as present in PM. High extracellular inorganic phosphate can alkalinize the cytosol (40), which in turn forces mitochondrial uptake of inorganic phosphate, resulting in enhanced mitochondrial superoxide generation (40–42). Mitochondrial superoxide represents the largest intracellular reservoir of reactive oxygen species (40). Thus, PM may induce oxidative stress-induced VIC damage. *In vivo*, high serum phosphate levels are known to induce AV calcification by stimulating complex intracellular signaling towards apoptosis and osteo-chondrogenic gene expression (43, 44). Oxidative stress and apoptosis thereby serve as initiators of calcific nodule formation in FCAVD (5, 45, 46). Conclusively, a phosphate-induced alkalization of the VICs cytosol with subsequent oxidative stress mediated apoptosis might be an underlying pathomechanism in patients with hyperphosphatemia, contributing to the initiation of ECM mineralization and calcific nodule formation. *In vitro*, VIC apoptosis has been described for osteogenic media containing *β*-glycerophosphate (12, 47).

Besides cell loss, media-dependent differences in cellular morphology were observed in our study. Whereas VICs in CM displayed heterogeneous cellular phenotypes, VICs in PM exhibited predominantly uniform spindle-like, thin, long cell bodies. In conjunction with the significant decrease of ACTA2 gene expression and abolished stress fiber assembly, one can hypothesize that PM-stimulated blocking of myofibroblastogenesis has occurred.

**Figure 5:**
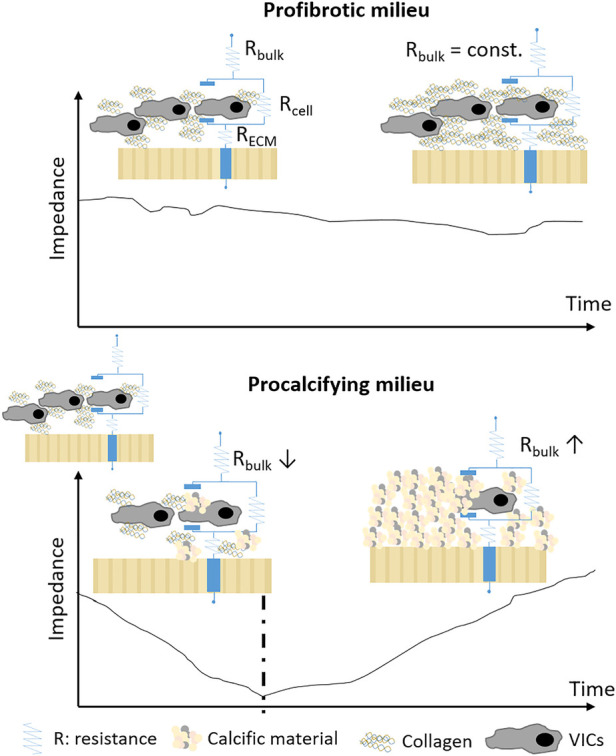
Scheme of EIS-based ECM mineralization detection. Modified using (48).

In summary, decline of myofibroblastogenesis, suppression of collagen synthesis, apoptosis and ECM mineralization create the PM-specific, biphasic EIS signal observed *in vitro* (Figure 5). In line with these results, the phenotype-specific cells that survived the apoptosis induce ECM mineralization. These processes have been described in and around calcific nodules in the AV leaflets *in vivo* (39). Therefore, VICs cultured in PM represent a suitable *in vitro* model of FCAVD, mimicking *in vivo*-like features in a reasonable time period.

### Pre-clinical and translational prospects of using high-sensitivity and high-throughput EIS in FCAVD exemplified by resolving sex-specific response to PM

EIS is preferable to standard methods, since it records changes in cellular electrical properties in a non-invasive environment and thereby allows real-time data acquisition of intra- and extracellular alterations due to external stimuli (49). The versatility of EIS was further demonstrated in our study when assessing sex- and donor- specific responses to PM. EIS demonstrated higher proliferative capacity of male VICs (see Supplementary Figure S2) with higher initial sensitivity to PM, indicating donor sex dependency. Additionally, EIS revealed a strong inter-individual heterogeneity of ECM mineralization *in vitro*, wherein strongest variability occurred in female VICs (see Supplementary Table S1). Further studies are required to determine whether this is a general characteristic and which hormonal status or comorbidities may underlie this observation. Finally, EIS analyses unveiled that individual VIC calcification potential and velocity *in vitro* are strongly donor specific.

## Data Availability

The original contributions presented in the study are included in the article/**Supplementary Material**, further inquiries can be directed to the corresponding author/s.
