# Supplementary material for: High resolution monitoring of valvular interstitial cell driven pathomechanisms in procalcific environment using label-free impedance spectroscopy
Source: Front Cardiovasc Med. 2023 Jun 20;10:1155371. doi: 10.3389/fcvm.2023.1155371 (PMC10319251; doi:10.3389/fcvm.2023.1155371)
Supplement: Supplementary file 1 [file Datasheet1.docx]

Supplementary Material

High resolution monitoring of valvular interstitial cell driven pathomechanisms in procalcific environment using label-free impedance spectroscopy

**Supplementary Figure 1.** Cell-free PM EIS measurement with and without collagen pre-coating. (a) Alizarin Red staining of E-Plates after 12 days of PM treatment. (b) EIS time profile over 12 days of PM treatment. PM: procalcifying medium. CI: cellular impedance.

**Supplementary Figure 2:** EIS based doubling time of native VICs in growth medium. Technical replicates are shown of n= 5 vs.5 donors. Student´s t-test.*p<0.05

**Supplementary Figure 3:** PM-specific EIS profiles of ten patients. All patients are showing a biphasic reaction to PM treatment. Please note that the scaling is adapted to the individual degree of severity. The dashed lines indicate the individual inflection points. CI: Cellular Impedance.

Supplementary Table 1: Recommended parameters for EIS - based characterization of calcification potential of large cohorts and outlier identification. PM- and patient-specific EIS characteristics. f: female, m: male, CI: cellular impedance

| Patient | sex | Phase 1 - slope  „sensitivity“ | time to minimal CI (days) | Phase 2 - slope  „remodeling“ | EIS-based  PM sensitivity |
| --- | --- | --- | --- | --- | --- |
| 1 | f | -0.45 ± 0.004 | 13 ± 0.6 | 0.05 ± 0.02 | moderate |
| 2 | f | *-0.25 ± 0.002* | *19 ± 0.3* | *0.001 ± 0.02* | *low* |
| 3 | f | -0.52 ± 0.004 | 8 ± 0.2 | 0.21 ± 0.04 | moderate |
| 4 | f | -0.97 ± 0.012 | 5 ± 0.04 | 1.85 ± 0.002 | high |
| 5 | f | -0.76 ± 0.011 | 6 ± 0.4 | 2.628 ± 0.05 | high |
| 6 | m | -0.93 ± 0.024 | 8 ± 0 | 0.128 ± 0.003 | moderate |
| 7 | m | -0.36 ± 0.023 | 8 ± 0.04 | 1.230 ± 0.02 | moderate |
| 8 | m | -0.53 ± 0.005 | 10 ± 0.3 | 0.209 ± 0.002 | moderate |
| 9 | m | -0.53 ± 0.013 | 7 ± 0.2 | 0.123 ± 0.002 | moderate |
| 10 | m | -0.41 ±0.003 | 12 ± 1.3 | 0.125 ± 0.002 | moderate |
